# Time to surgery is not an oncological risk factor in HCC patients undergoing liver resection

**DOI:** 10.1007/s00423-023-02922-4

**Published:** 2023-05-10

**Authors:** Carlos Constantin Otto, Guanwu Wang, Anna Mantas, Daniel Heise, Philipp Bruners, Sven Arke Lang, Tom Florian Ulmer, Ulf Peter Neumann, Lara Rosaline Heij, Jan Bednarsch

**Affiliations:** 1 Department of Surgery and Transplantation, University Hospital RWTH Aachen, Pauwelsstrasse 30, 52074 Aachen, Germany; 2 Department of Radiology, University Hospital RWTH Aachen, Aachen, Germany; 3 Department of Surgery, Maastricht University Medical Centre (MUMC), Maastricht, Netherlands; 4 Institute of Pathology, Maastricht University Medical Centre (MUMC), Maastricht, Netherlands

**Keywords:** HCC, Time-to-surgery, Surgery, Recurrence, Overall survival

## Abstract

**Purpose:**

Given limitations of the health care systems in case of unforeseeable events, e.g., the COVID pandemic as well as trends in prehabilitation, time from diagnosis to surgery (time to surgery, (TTS)) has become a research issue in malignancies. Thus, we investigated whether TTS is associated with oncological outcome in HCC patients undergoing surgery.

**Methods:**

A monocentric cohort of 217 patients undergoing liver resection for HCC between 2009 and 2021 was analyzed. Individuals were grouped according to TTS and compared regarding clinical characteristics. Overall survival (OS) and recurrence-free survival (RFS) was compared using Kaplan-Meier analysis and investigated by univariate and multivariable Cox regressions.

**Results:**

TTS was not associated with OS (*p*=0.126) or RFS (*p*=0.761) of the study cohort in univariate analysis. In multivariable analysis age (*p*=0.028), ASA (*p*=0.027), INR (0.016), number of HCC nodules (*p*=0.026), microvascular invasion (MVI; *p*<0.001), and postoperative complications (*p*<0.001) were associated with OS and INR (*p*=0.005), and number of HCC nodules (*p*<0.001) and MVI (*p*<0.001) were associated with RFS. A comparative analysis of TTS subgroups was conducted (group 1, ≤30 days, *n*=55; group 2, 31–60 days, *n*=79; group 3, 61–90 days, *n*=45; group 4, >90 days, *n*=38). Here, the median OS were 62, 41, 38, and 40 months (*p*=0.602 log rank) and median RFS were 21, 26, 26, and 25 months (*p*=0.994 log rank). No statistical difference regarding oncological risk factors were observed between these groups.

**Conclusion:**

TTS is not associated with earlier tumor recurrence or reduced overall survival in surgically treated HCC patients.

**Supplementary Information:**

The online version contains supplementary material available at 10.1007/s00423-023-02922-4.

## Introduction

Hepatocellular carcinoma (HCC) is a major global health burden contributing notably to the worldwide cancer-related mortality [1, 2]. While systemic and interventional therapies, e.g., trans-arterial chemoembolization (TACE) or radiofrequency ablation (RFA), are the main options in advanced tumor stages, liver resection remains the gold standard in earlier stages with preserved liver function [3]. Proper patient selection and the implantation of modern liver function assessment as well as minimal invasive liver resection did further allow to widen the patient population eligible for surgery improving outcome in individuals formerly treated by TACE or local ablative procedure [4–7]. While liver transplantation remains the treatment of choice in terms of recurrence for localized HCC, strict allocation rules and the limited availability of donor grafts preclude transplantation for a large proportion of HCC patients [8]. Therefore, liver resection is becoming increasingly popular across the whole spectrum of localized HCC requiring medical resources, e.g., surgical as well as intensive care capacity to conduct surgery in these patients [9, 10].

During the last 2 years, the global health systems have shifted resources to encounter the COVID-19 pandemic. Thus, curative intention surgery in oncological patients was frequently delayed and the corresponding impact on clinical outcome investigated [11]. Reduced overall survival (OS) of patients with different malignant diseases due to delayed time to surgery (TTS) in the scenario of surgical, systemic (adjuvant, neoadjuvant), and radiotherapy has been described [12]. Interestingly, in a recent international study of colorectal cancer patients, neither poorer outcomes nor compromised resectability was observed after a treatment delay during the COVID pandemic [13]. However, the role of TTS in the oncological outcome of HCC patients remains to be elucidated. Thus, the aim of this study was to investigate the impact of TTS on short- and long-term outcome in HCC patients.

## Material and methods

### Patients

Between 2009 and 2021, 240 patients underwent liver resection for HCC at the University Hospital RWTH Aachen (UH-RWTH). Of these, patients who underwent any neoadjuvant therapy (*n*=15) were treated as emergency cases due to active bleeding (n=3) and those who had no images in the radiological archives (*n*=5) were excluded from the study. As such, two hundred seventeen (*n*=217) patients were eligible for the TTS analysis (Supplementary Figure 1). All patients underwent detailed, internationally accepted staging. Therefore, only patients with localized HCC without distant metastasis were analyzed. The study was conducted at the UH-RWTH in accordance with the requirements of the Institutional Review Board of the RWTH-Aachen University (EK 22-342), the current version of the Declaration of Helsinki, and the good clinical practice guidelines (ICH-GCP).

### Study definitions

TTS was calculated as the date difference from the date of diagnosis to the date of surgery. The date of diagnosis was set as the date of the first contrast-enhanced ultrasound, magnetic resonance imaging (MRI), or computed tomography (CT) indicating the presence of HCC. Imaging data of our center as well as external referring hospitals were used for this analysis depending on first hospital site of diagnosis. All imaging modalities were evaluated for diagnostic quality by a senior radiologist (PB).

### Staging and surgical technique

Preoperatively, all patients were evaluated for general performance, operability, and liver function as previously described [14, 15]. Standard staging procedures were carried out by means of MRI or CT to define tumor burden and exclude distant metastasis. American society of anesthesiologists (ASA) and Eastern Cooperative Oncology Group (ECOG) performance status were used to evaluate the physical status of patients. Liver function was evaluated by standard laboratory parameters and the LiMAx test (Humedics® GmbH, Berlin, Germany) [7].

Patients staged Barcelona Clinic Liver Cancer (BCLC) A to C without signs of extrahepatic tumor burden and preserved liver function were considered to be surgical candidates and discussed within the institutional interdisciplinary tumor board. The indication for hepatectomy was finally made by an experienced hepatobiliary surgeon. In cases of HCC recurrence, operative resection was discussed within an interdisciplinary tumor board in cases with localized disease evaluating ECOG status, tumor morphology, and residual liver function. Patients considered no surgical candidates were referred to interventional therapies (TACE, RFA, microwave ablation, stereotactic radiation), systemic therapy, or best supportive care with respect to common international guidelines [9, 10].

Liver resection was carried out as described previously and carried out in accordance with department-specific surgical standards in every case [14, 15]. Intraoperatively, ultrasound was used to visualize tumor spread and exclude additional suspect lesions. For transection of liver parenchyma in open surgery, the Cavitron Ultrasonic Surgical Aspirator (CUSA®, Integra LifeSciences®, Plainsboro NJ, USA) was used. To avoid blood loss, low central venous pressure was maintained during transection and intermittent Pringle maneuvers were used if necessary. For parenchymal transection in laparoscopic hepatectomy, Thunderbeat ® (Olympus K.K., Tokyo, Japan), Harmonic Ace ® (Ethicon Inc. Somerville, NJ, USA), or laparoscopic CUSA (Integra life sciences, New Jersey, USA) in combination with vascular staplers (Echelon, Ethicon, Somerville, New Jersey, USA) or polymer clips (Teleflex Inc., Pennsylvania, USA) were preferably used.

### Statistical analysis

The primary objective of this study was to investigate the oncological effect of TTS on OS and recurrence-free survival (RFS) in HCC patients undergoing surgical resection. OS was defined as the period from date of liver resection to the date of death from any cause or date of the last contact if the patient was alive. RFS was defined as the period from liver resection to the date of recurrence. Patients with no tumor recurrence were censored at date of death or at the last follow-up for RFS analysis. For group comparison, patient subgroups with respect to TTS were generated (1–30 days, 31–60 days, 61–90 days, and over 90 days). Chi-square test was used to compare categorial data, expressed with number and percentage. Continuous variables were expressed as median and interquartile range and compared by Kruskal-Wallis test. A *p* value <0.05 was considered to indicate statistical significance. Kaplan-Meier analysis was used to generate survival curves. Univariate cox regression was to determine variables associated with OS and RFS. Significant parameters (*p*<0.05) were proceeded to a multivariable cox regression model and analyzed within a backward selection. Median follow-up was assessed with the reverse Kaplan-Meier method. Complications are reported using the Clavien-Dindo scale [16]. Perioperative mortality (Clavien-Dindo V) was defined as in-hospital mortality. All data processing was conducted by SPSS Statistics 24 (IBM Corp., Armonk, NY, USA).

## Results

### Patient cohort

A total of 217 patients underwent liver resection for HCC in curative intention from 2009 to 2021 at our institution were included in this study. Most of the patients were male (71.4%), the median age in the overall cohort was 69 years. A major part of the cohort (65%) displayed an ASA score of III and more. Alcohol-induced (23.5%) and non-alcoholic fatty liver disease (26.9%) along with viral induced hepatitis (24.9%) were the main underlying liver diseases in the cohort; a subset of patients (14.7%) presented with either cryptogenic or a less common liver disease (e.g., hemochromatosis). The largest proportion of the cohort (56.7%) was BCLC stage A at time of resection, whereas a subset of patients was categorized CHILD Pugh B (8.3%). The median number of HCC nodules was 1 (interquartile range: 1–2) with a median diameter of 50 mm (interquartile range: 33–80) of the largest lesion. Also, a notable proportion of patients (25.8%) showed macrovascular invasion in preoperative imaging. The median operative time was 204 min (interquartile range: 137–270) and the most common operative procedure was atypical liver resection (37.3%), followed by left/right hepatectomy (22.6%). Red blood cells (24.4%) and fresh frozen plasma (FFP) (36.4%) were administered intraoperatively on demand. R0 resection was achieved in most cases (94.5%; reasons for R1 resection presented in Supplementary Table 1). Of all individuals, 24.5% experienced complications Clavien-Dindo > II and 5.1% of the cohort deceased during hospitalization (reasons for perioperative mortality presented in Supplementary Table 2). Detailed perioperative characteristics are depicted in Table 1.

**Table 1:** Study cohort

Variables	HCC cohort (*n*=217)
Demographics	
Gender, m/f (%)	155 (71.4)/62 (28.6)
Age (years)	69 (60.5–76)
BMI (kg/m^2^)	26.2 (23.3–29.4)
ASA, *n* (%)	
I	2 (0.9)
II	74 (34.1)
III	135 (62.2)
IV	6 (2.8)
V	0
Liver disease, *n* (%)	
ALD	51 (23.5)
NAFLD	80 (26.9)
Viral	54 (24.9)
Cryptogenic/others	32 (14.7)
Preoperative liver function	
MELD score	6 (6–6.7)
AFP (ng/ml)	9 (3.4–88.7)
Albumin (g/dl)	4 (3.6–4.4)
AST (U/l)	39 (26–56)
ALT (U/l)	33 (22–52)
GGT (U/l)	97 (53–199)
Total bilirubin (mg/dl)	0.56 (0.4–0.8)
Platelet count (/nl)	211 (163–272)
Alkaline phosphatase (U/l)	101 (75–139)
Prothrombin time (%)	93 (85–100)
INR	1.04 (0.98–1.11)
Creatinine (mg/dl)	0.87 (0.7–1.06)
Hemoglobin (g/dl)	13.3 (11.7–14.7)
Child Pugh, *n* (%)	
A	199 (91.7)
B	18 (8.3)
C	0
Child Pugh score	5
Preoperative imaging features	
Number of nodules	1 (1–2)
Largest nodule diameter (mm)	50 (33–80)
Tumor burden > 50%, *n* (%)	9 (4.1)
Overall macrovascular invasion, *n* (%)	56 (25.8)
Portal vein invasion, *n* (%)	37 (17.1)
Extrahepatic vascular invasion, *n* (%)	12 (5.5)
Portal vein thrombosis, *n* (%)	11 (5.1)
Ascites, *n* (%)	8 (3.7)
BCLC, *n* (%)	
0	11 (5.1)
A	123 (56.7)
B	45 (20.7)
C	37 (17.1)
Operative data	
Laparoscopic resection, *n* (%)	75 (34.6)
Conversion rate, *n* (%)	5 (2.3)
Reason for conversion, *n* (%)	
Intraoperative hemorrhage	3 (1.4)
Technical considerations	2 (0.9)
Operative time (minutes)	204 (137–270)
Operative procedure, *n* (%)	
Atypical	81 (37.3)
Segmentectomy	30 (13.8)
Bisegmentectomy	19 (8.8)
Hemihepatectomy	49 (22.6)
Extended liver resection	28 (12.9)
ALPPS/TSH	8 (3.7)
Other*	2 (0.9)
Additional procedures**, *n* (%)	12 (5.5)
Intraoperative blood transfusion, *n* (%)	53 (24.4)
Intraoperative FFP, *n* (%)	79 (36.4)
Intraoperative platelet transfusion, *n* (%)	2 (0.9)
Pathological examination	
R0 resection, *n* (%)	205 (94.5)
T category, *n* (%)	
T1	94 (43.3)
T2	79 (36.4)
T3/T4	43 (19.8)
Microvascular invasion, *n* (%)	81 (37.3)
Tumor grading, *n* (%)	
G1	10 (4.6)
G2	162 (74.7)
G3/G4	40 (18.4)
Postoperative Data	
Intensive care stay, days	1
Hospitalization, days	8 (6–14)
Postoperative complications, *n* (%)	
No complications	108 (49.8)
Clavien-Dindo I	22 (10.1)
Clavien-Dindo II	34 (15.7)
Clavien-Dindo IIIa	21 (9.7)
Clavien-Dindo IIIb	10 (4.6)
Clavien-Dindo IVa	10 (4.6)
Clavien-Dindo IVb	1 (0.5)
Clavien-Dindo V	11 (5.1)
PHLF 50-50 criteria***, *n* (%)	3 (1.4)
PHLF ISGLS***, *n* (%)	40 (18.4)
ISGLS Grade, *n* (%)	
A	27 (12.4)
B	7 (3.2)
C	6 (2.8)
Postoperative blood transfusion, *n* (%)	37 (17.1)
Postoperative FFP, *n* (%)	14 (6.5)
Postoperative platelet transfusion	7 (3.2)
Follow-up data	
Recurrence-free survival (months)	26 (19–33)
Overall survival (months)	42 (30–54)

Data presented as median and interquartile range if not noted otherwise. Follow-up data is presented as median and 95% CI

*ALD* alcoholic liver disease, *ALT* alanine aminotransferase, *ASA* American Society of Anesthesiologists Classification, *AST* aspartate aminotransferase, *BCLC* Barcelona Clinical Liver Cancer Staging System, *BMI* body mass index, *FFP* fresh frozen plasma, *GGT* gamma glutamyltransferase, *INR* international normalized ratio, *MELD* model of end-stage liver disease, *NAFLD* non-alcoholic fatty liver disease, *PHLF* post-hepatectomy liver failure

*Other procedures summarize operations which are not described within the standard reporting system (e.g., multiple atypical resections/combination of various anatomical and atypical resection)

**Additional procedures refer to radiofrequency and microwave ablation to achieve complete tumor clearance

***Postoperative liver failure was assessed by the 50-50 criteria and the ISGLS definition [43, 44]

### Time-to-surgery with respect to different characteristics

Interestingly, the median TTS in the overall cohort was 49 days (interquartile range (IQR): 30–83). No statistical difference in TTS between patients diagnosed in our center (21.2%, 54 days (IQR: 35–84)) and externally diagnosed patients (78.8%, 47 days (IQR: 27–79)) has been found (*p*=0.15). Patients treated during the COVID period from year 2020 to 2021 (27.2%) had a median TTS of 70 days (IQR: 42–90), resulting to a statistically significant longer TTS than patients treated before 2020 (72.8%, 46 days (IQR: 24–73)) (*p*<0.001).

### Comparative analysis of the patient cohort

Categorized by time to surgery, 55 patients underwent liver resection within 30 days after diagnosis, 79 patients between 31 and 60 days, 45 between 61 and 90 days, and 38 patients after 90 days. Extensive group comparisons revealed no differences in major demographic and oncological characteristics. Differences were observed in gender (*p*=0.020) and largest tumor diameter (*p*=0.020) with this difference being based on larger tumors in TTS 1–30 days group compared to TTS 61–90 days (*p*=0.004) and TTS > 90 days (*p*=0.015) group. Furthermore, the distribution of laparoscopic resections differed significantly between the subgroups (*p*=0.001). Other examined parameters showed no statistical differences in distribution, detailed perioperative results for the 4 subgroups are described in Table 2.

**Table 2:** Comparative analysis of patients undergoing liver resection for hepatocellular carcinoma

Variables	Time-to-surgery analysis				
	TTS 1–30 days (*n*=55)	TTS 31–60 days (*n*=79)	TTS 61–90 days (*n*=45)	TTS > 90 days (*n*=38)	*p*-value
Demographics					
Gender, m/f (%)	33 (60)/22 (40)	55 (69.6)/24 (30.4)	33 (73.3)/12 (26.7)	34 (89.5)/4 (10.5)	**0.020**
Age (years)	68 (60–75)	69 (60–75)	72 (61–77)	72 (63–76)	0.525
BMI (kg/m^2^)	26.6 (23.6–30.4)	25.6 (23.1–29.3)	26.2 (22.9–31.4)	26.3 (24–30)	0.867
ASA, *n* (%)					0.199
I	0	1 (1.3)	0	1 (2.6)	
II	24 (43.6)	23 (29.1)	18 (40)	9 (23.7)	
III	28 (50.9)	54 (68.4)	25 (55.6)	28 (73.7)	
IV	3 (5.5)	1 (1.3)	2 (4.4)	0	
V	0	0	0	0	
Liver disease, *n* (%)					0.181
ALD	6 (10.9)	25 (31.6)	12 (26.7)	8 (21.1)	
NAFLD	22 (40)	27 (34.2)	16 (35.6)	15 (39.5)	
Viral	16 (29.1)	14 (17.7)	14 (31.1)	10 (26.3)	
Cryptogenic/others	11 (20)	13 (16.5)	3 (6.7)	5 (13.2)	
Preoperative liver function					
MELD score	6	6 (6–6.9)	6 (6–6.9)	6 (6–7.2)	0.292
AFP (ng/ml)	6.8 (2.5–561.6)	12.1 (3.9–63.4)	8 (3.5–18)	7.6 (4.2–102.5)	0.723
Albumin (g/dl)	4 (3.7–4.4)	4 (3.6–4.4)	4 (3.7–4.5)	4.2 (3.7–4.5)	0.441
AST (U/l)	41.5 (31.5–65)	38 (24.8–58)	35 (25–54.5)	39 (23.5–53.8)	0.303
ALT (U/l)	40 (25–60)	32 (20.3–54.5)	30 (23.8–45.5)	33 (20.5–52.3)	0.233
GGT (U/l)	88.5 (57.3–190.3)	109 (51–211)	95 (54–217)	108 (50–184)	0.965
Total bilirubin (mg/dl)	0.52 (0.38–0.73)	0.58 (0.4–0.8)	0.61 (0.41–0.82)	0.57 (0.42–0.87)	0.359
Platelet count (/nl)	237 (189–305)	202 (150–262)	215 (167–264)	200 (134–263)	0.082
Alkaline phosphatase (U/l)	102 (76–140)	101 (67–139)	101 (75–144)	101 (78–137)	0.971
Prothrombin time (%)	98 (88–103)	91 (78–100)	91 (82–101)	94 (87–99)	0.122
INR	1.01 (0.96–1.08)	1.06 (0.99–1.12)	1.06 (0.99–1.12)	1.04 (1.01–1.1)	0.161
Creatinine (mg/dl)	0.85 (0.7–1.06)	0.86 (0.7–1.09)	0.9 (0.73–1.06)	0.87 (0.76–1.06)	0.718
Hemoglobin (g/dl)	13.3 (12–14.7)	13 (11.5–14.5)	13.6 (11.7–14.9)	13.2 (11.7–14.6)	0.548
Child Pugh, *n* (%)					0.109
A	52 (94.5)	68 (86.1)	44 (97.8)	35 (92.1)	
B	3 (5.5)	11 (13.9)	1 (2.2)	3 (7.9)	
C	0	0	0	0	
Child Pugh score	5	5 (5–6)	5	5 (5–6)	0.288
Preoperative imaging features					
Number of nodules	1 (1–2)	1 (1–2)	1 (1–2)	1 (1–2)	0.748
Largest nodule diameter (mm)	65 (43–100)	47 (32–81)	42 (27.5–58)	49.5 (30.8–71.3)	**0.020**
Tumor burden > 50%, *n* (%)	4 (7.3)	3 (3.8)	2 (4.4)	0	0.396
Overall macrovascular invasion, *n* (%)	20 (36.4)	18 (22.8)	11 (24.4)	7 (18.4)	0.195
Portal vein invasion, *n* (%)	13 (23.6)	13 (16.5)	7 (15.6)	4 (10.5)	0.404
Extrahepatic vascular invasion, *n* (%)	4 (7.3)	3 (3.8)	2 (4.4)	3 (7.9)	0.733
Portal vein thrombosis, *n* (%)	4 (7.3)	4 (5.1)	2 (4.4)	1 (2.6)	0.787
Ascites, *n* (%)	1 (1.8)	4 (5.1)	0	3 (7.9)	0.205
BCLC, *n* (%)					0.899
0	3 (5.5)	5 (6.3)	2 (4.4)	1 (2.6)	
A	29 (52.7)	43 (54.4)	26 (57.8)	25 (65.8)	
B	10 (18.2)	17 (21.5)	10 (22.2)	8 (21.1)	
C	13 (23.6)	13 (16.5)	7 (15.6)	4(10.5)	
Operative data					
Laparoscopic resection, *n* (%)	7 (12.7)	29 (36.7)	20 (44.4)	19 (50)	**0.001**
Conversation rate, *n* (%)	1 (1.8)	2 (2.5)	1 (2.2)	1 (2.6)	0.992
Operative time (minutes)	220 (150–269)	208 (135–292)	194 (117–265)	200 (144–262)	0.426
Operative procedure, *n* (%)					0.053
Atypical	9 (16.4)	32 (40.5)	20 (44.4)	20 (52.6)	
Segmentectomy	6 (10.9)	12 (15.2)	8 (17.8)	4 (10.5)	
Bisegmentectomy	6 (10.9)	6 (7.6)	3 (6.7)	4 (10.5)	
Hemihepatectomy	18 (32.7)	19 (24.1)	8 (17.8)	4 (10.5)	
Extended liver resection	14 (25.5)	6 (7.6)	4 (8.9)	4 (10.5)	
ALPPS/TSH	0	1 (1.3)	1 (2.2)	0	
Other*	2 (3.6)	3 (3.8)	1 (2.2)	2 (5.3)	
Additional procedures**, *n* (%)	1 (1.8)	6 (7.6)	3 (6.7)	2 (5.3)	0.530
Intraoperative blood transfusion, *n* (%)	17 (30.9)	21 (26.6)	9 (20)	6 (15.8)	0.340
Intraoperative FFP, *n* (%)	26 (47.3)	29 (36.7)	12 (26.7)	12 (31.6)	0.162
Intraoperative platelet transfusion, n (%)	0	1 (1.3)	0	1 (2.6)	0.504
Pathological examination					
R0 resection, *n* (%)	52 (94.5)	76 (96.2)	42 (93.3)	35 (92.1)	0.969
T category, *n* (%)					0.073
T1	24 (43.6)	33 (41.8)	25 (55.6)	12 (31.6)	
T2	15 (27.3)	32 (40.5)	12 (26.7)	20 (52.6)	
T3/T4	16 (29.1)	13 (16.5)	8 (17.8)	6 (15.8)	
Microvascular invasion, *n* (%)	24 (43.6)	27 (34.2)	15 (33.3)	15 (39.5)	0.548
Tumor grading, *n* (%)					0.743
G1	2 (3.6)	4 (5.1)	1 (2.2)	3 (7.9)	
G2	43 (78.2)	55 (69.6)	37 (82.2)	27 (71.1)	
G3/G4	10 (18.2)	17 (21.5)	6 (13.3)	7 (18.4)	
Postoperative data					
Intensive care stay, days	1	1	1	1	0.766
Hospitalization, days	10 (7–14)	8 (5–14)	8 (6–15)	8 (5–13)	0.422
Postoperative complications, *n* (%)					0.866
No complications	25 (45.5)	42 (53.2)	23 (51.1)	18 (47.4)	
Clavien-Dindo I	7 (12.7)	7 (8.9)	5 (11.1)	3 (7.9)	
Clavien-Dindo II	9 (16.4)	10 (12.7)	8 (17.8)	7 (18.4)	
Clavien-Dindo IIIa	6 (10.9)	9 (11.4)	3 (6.7)	3 (7.9)	
Clavien-Dindo IIIb	5 (9.1)	2 (2.5)	2 (4.4)	1 (2.6)	
Clavien-Dindo IVa	1 (1.8)	5 (6.3)	1 (2.2)	3 (7.9)	
Clavien-Dindo IVb	1 (1.8)	0	0	0	
Clavien-Dindo V	1 (1.8)	4 (5.1)	3 (6.7)	3 (7.9)	
PHLF 50-50 criteria***, *n* (%)	0	1 (1.3)	0	2 (5.3)	0.130
PHLF ISGLS***, *n* (%)	7 (12.7)	17 (21.5)	7 (15.6)	9 (23.7)	0.439
ISGLS grade, *n* (%)					0.472
A	5 (9.1)	12 (15.2)	6 (13.3)	4 (10.5)	
B	2 (3.6)	2 (2.5)	0	3 (7.9)	
C	0	3 (3.8)	1 (2.2)	2 (5.3)	
Postoperative blood transfusion	17 (30.9)	21 (26.6)	9 (20)	6 (15.8)	0.340
Postoperative FFP transfusion	3 (5.5)	5 (6.3)	3 (6.7)	3 (7.9)	0.965
Postoperative platelet transfusion	2 (3.6)	2 (2.5)	1 (2.2)	2 (5.3)	0.829
Follow-up data					
Recurrence-free survival (months)	21 (11–31)	26 (6–46)	26 (14–38)	25 (18–32)	0.994
Overall survival (months)	62 (22–102)	41 (19–63)	38 (21–55)	40 (15–65)	0.602

Data presented as median and interquartile range if not noted otherwise. Follow-up data is presented as median and 95% CI. Chi-square test was used to compare categorial data, expressed with number and percentage. Continuous variables were expressed as median and interquartile range and compared by Kruskal-Wallis test. For statistically significant parameters (*p*<0.05) bold entries were used

*ALD* alcoholic liver disease, *ALT* alanine aminotransferase, *ASA* American Society of Anesthesiologists Classification, *AST* aspartate aminotransferase, *BCLC* Barcelona Clinical Liver Cancer Staging System, *BMI* body mass index, *FFP* fresh frozen plasma, *GGT* gamma glutamyltransferase, *INR* international normalized ratio, *MELD* model of end-stage liver disease, *NAFLD* non-alcoholic fatty liver disease, *PHLF* post-hepatectomy liver failure

*Other procedures summarize operations which are not described within the standard reporting system (e.g., multiple atypical resections/combination of various anatomical and atypical resection)

**Additional procedures refer to radiofrequency and microwave ablation to achieve complete tumor clearance

***Postoperative liver failure was assessed by the 50-50 criteria and the ISGLS definition [43, 44]

### Survival analysis

**Fig. 1:**
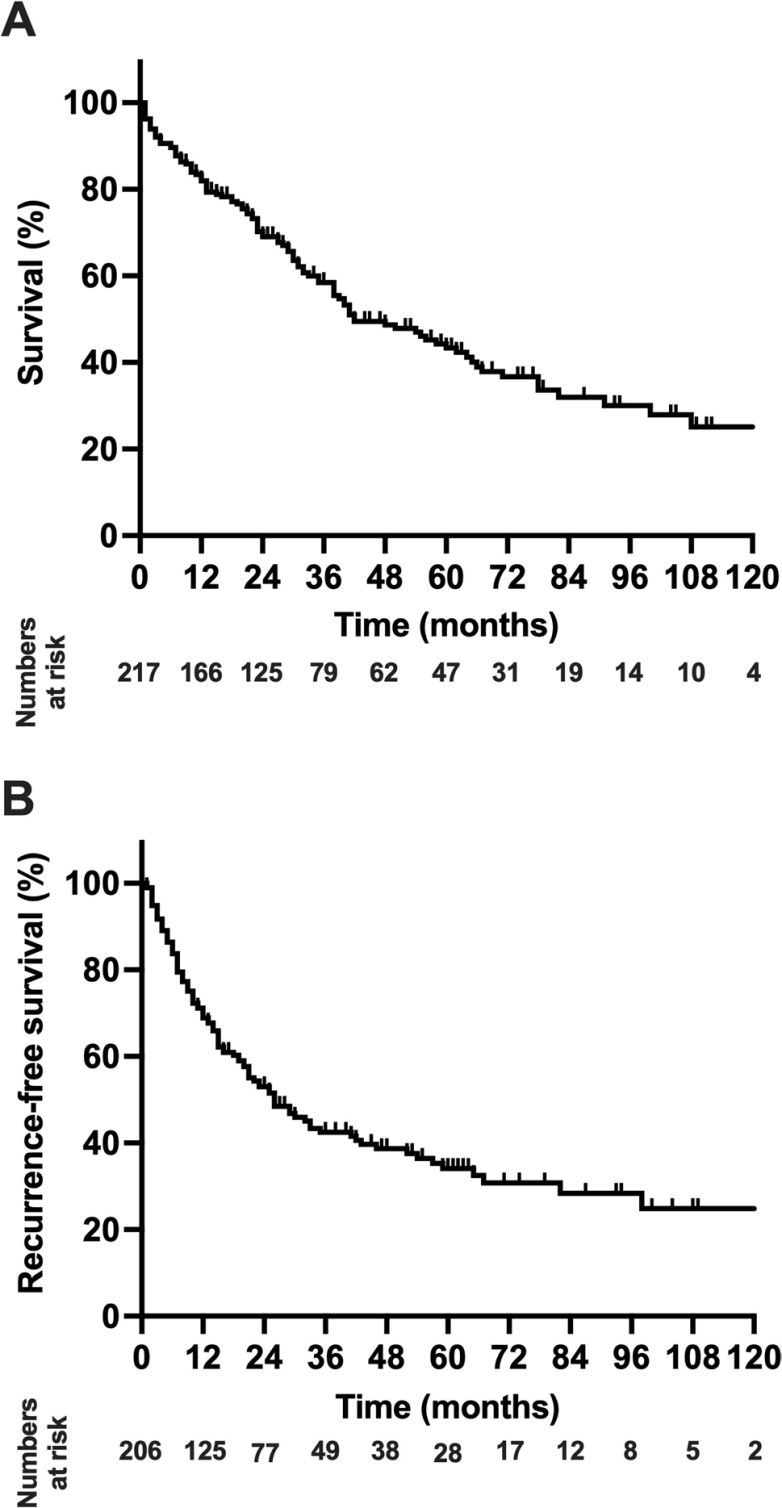
Oncological survival in hepatocellular carcinoma of the study cohort. **A** Overall survival. The median OS of the cohort was 42 months. **B** Recurrence-free survival. The median RFS of the cohort was 26 months. OS, overall survival; RFS, recurrence-free survival

**Fig. 2:**
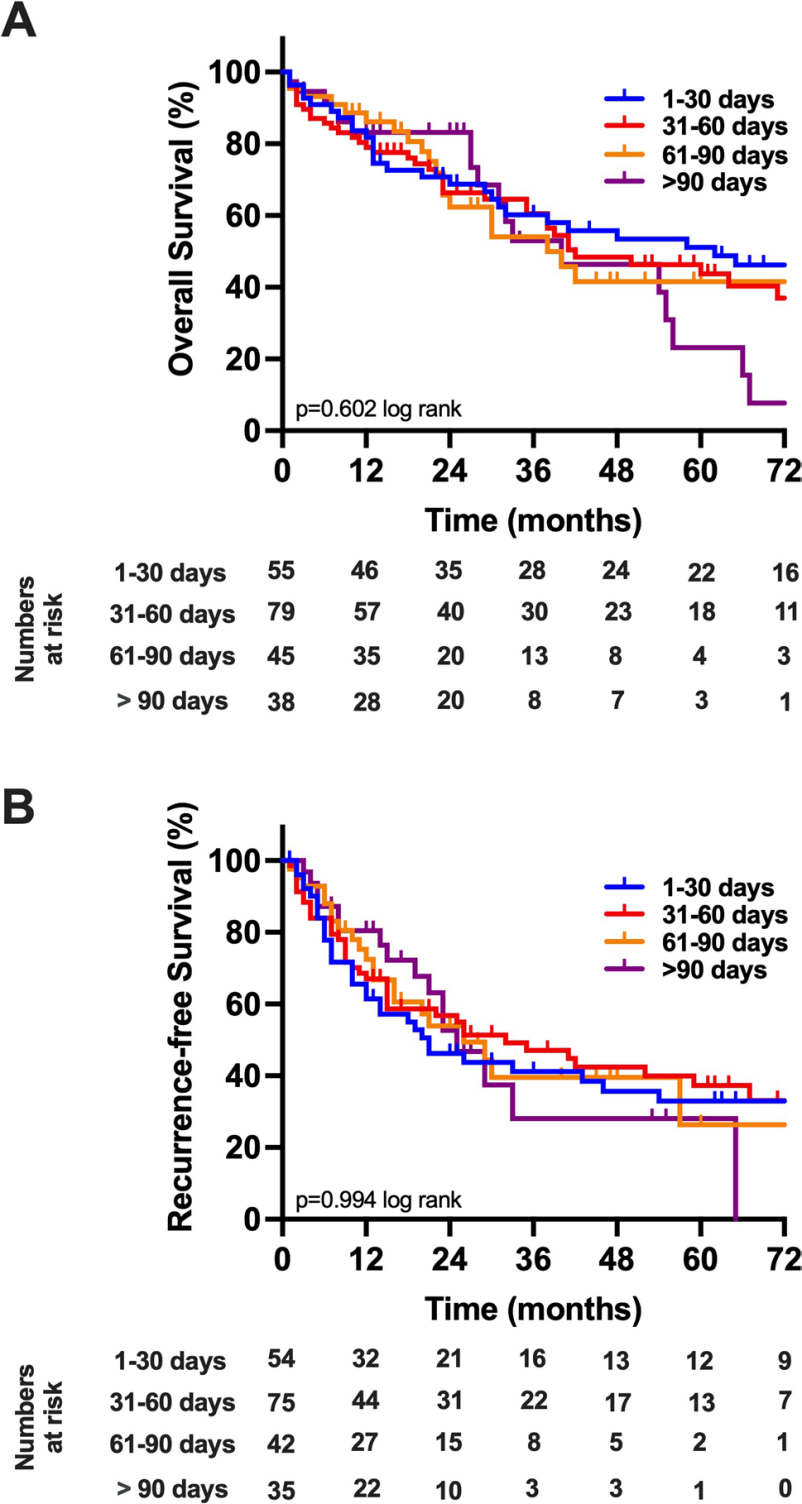
Oncological survival in hepatocellular carcinoma stratified by time to surgery. **A** Overall survival. The median OS was 62 in patients with a TTS less than 31 days, while the median OS was 41 months in patients with a TTS between 31 and 60 days, 38 months in patients with a TTS between 61 and 90 days, and 40 months in patients with a TTS more than 90 days (*p*=0.602 log rank). **B** Recurrence-free survival. The median RFS was 21 months (95% CI: 11–31 months) in patients with a TTS less than 31 days, 26 months in patients with a TTS between 31 and 60 days, 26 months in patients with a TTS between 61 and 90 days, and 25 months in patients with a TTS more than 90 days (*p*=0.994 log rank)

After a median follow-up of 59 months, the median OS of the cohort was 42 months (95% CI: 30–54 months; 3-year OS=58%, 5-year OS=43%) and the median RFS was 26 months (95% CI: 19–33 months; 3-year RFS=42%, 5-year RFS=32%; Fig. 1). Regarding the analysis investigating TTS, the median OS was 62 months (95% CI: 22–102 months) in patients with a TTS less than 31 days, while the median OS was 41 months (95% CI: 19–63 months) in patients with a TTS between 31 and 60 days, 38 months (95% CI: 21–55 months) in patients with a TTS between 61 and 90 days, and 40 months (95% CI: 15–64 months) in patients with a TTS more than 90 days (*p*=0.602 log rank, Fig. 2A). For RFS analysis, 5 patients were excluded from RFS analysis due to missing recurrence data. Here, no difference in RFS was detected regarding TTS, with a median RFS of 21 months (95% CI: 11–31 months) in patients with a TTS less than 31 days, a median RFS was 26 months (95% CI: 6–46 months) in patients with a TTS between 31 and 60 days, 26 months (95% CI: 14–38 months) in patients with a TTS between 61 and 90 days, and 25 months (95% CI: 18–32 months) in patients with a TTS more than 90 days (*p*=0.994 log rank, Fig. 2B).

### Univariate and multivariable Cox regressions

Cox regressions were used for OS and RFS to identify risk factors for impaired oncological outcomes*.* For OS, gender (*p*=0.002), age (*p*=0.031), ASA score (<0.001), MELD (*p*=0.002) and CHILD Pugh Score (*p*=0.005), and INR (*p*=0.001) as well as various other liver function parameters, number of nodules (*p*<0.001), and largest nodule diameter (*p*=0.013) next to various other preoperative imaging features, laparoscopic resection (*p*=0.001), additional procedures to resection (*p*=0.045), intraoperative red blood cells (*p*<0.001) and FFP (*p=*0.010) transfusion, R1 resection (*p*=0.012), pT category (*p*<0.001), microvascular invasion (MVI, *p*<0.001), and postoperative duration of hospitalization (*p*=0.014) and complications (*p*<0.001) as well as postoperative transfusion of red blood cells (*p*=0.047) and FFP (*p*=0.046) gained statistical significance in univariate analysis (Table 3). Subsequently, those parameters were transferred to multivariable analysis (194 patients (89.4%) included due to data availability). In here, age (*p*=0.009), ASA score (*p*=0.012), INR (*p*=0.008), number of nodules (*p*=0.017), MVI (*p*=0.016), and postoperative complications (*p*<0.001) were identified as independent predictors for OS (Table 3). TTS showed no statistical significance in OS (*p*=0.126). A similar approach was conducted for RFS (183 patients (91.0%) included due to data availability). Comparable to OS, some preoperative liver function values and various preoperative imaging features as well as R1 resection (*p*=0.018), pT category (*p*<0.001), and MVI (*p*<0.001) showed statistical significance in univariate analysis. Subsequently, a multivariable Cox regression was carried out with those parameters. Here, INR (*p*=0.011), number of nodules (*p*<0.001), and MVI (*p*<0.001) were independent prognostic factors for RFS (Table 4). As in OS, TTS was no relevant prognostic factor for RFS (*p*=0.759).

**Table 3:** Univariate and multivariable analysis of overall survival in hepatocellular carcinoma

	Univariate analysis		Multivariable analysis	
	HR (95% CI)	*p* value	HR (95% CI)	*p* value
Demographics				
Gender (male=1)	2.02 (1.28–3.17)	**0.002**	1.46 (0.87–2.46)	0.153
Age (years)	1.02 (1–1.04)	**0.031**	1.02 (1.01–1.04)	**0.028**
BMI (≤25 kg/m^2^=1)	1.18 (0.8–1.74)	0.400		
ASA (I/II=1)	2.14 (1.4–3.28)	**<0.001**	1.74 (1.07–2.84)	**0.027**
Liver disease		0.141		
ALD	1			
NAFLD	0.77 (0.48–1.24)			
Viral	0.69 (0.41–1.16)			
Cryptogenic/others	0.44 (0.22–0.91)			
Time-to-surgery		0.608		
1–30 days	1			
31–60 days	1.17 (.73–1.88)			
61–90 days	1.28 (.73–2.24)			
>90 days	1.47 (.82–2.64)			
Time-to-surgery (quantitatively)	1.01 (1.00–1.01)	0.126		
Preoperative liver function				
MELD score (under 6 =1)	1.13 (1.04–1.19)	**0.002**	1.03 (0.94–1.13)	0.492
Albumin (g/l)	0.52 (0.37–.72)	**<0.001**	0.82 (0.55–1.2)	0.305
AFP (μg/l)	1 (0.99–1.01)	**0.001**	excl.*	
AST (U/l)	1.01(1–1.01)	0.077		
ALT (U/l)	1 (0.99–1.01)	0.582		
GGT (U/l)	1.01 (1–1.02)	**0.002**	0.99 (0.98–1.00)	0.322
Bilirubin (mg/dl)	1.55 (1.12–2.15)	**0.008**	0.85 (0.56–1.30)	0.447
AP (U/l)	1 (0.99–1.01)	0.446		
Platelet count (/nl)	1 (0.99–1.01)	0.983		
INR	24.73 (3.83–159.8)	**0.001**	11.87 (1.58–88.97)	**0.016**
Creatinine (mg/dl)	0.77 (0.46–1.26)	0.297		
Hemoglobin (g/dl)	0.96 (0.86–1.07)	0.436		
Child Pugh (A=1)	2.31 (1.26–4.22)	**0.005**	0.84 (0.29–2.39)	0.741
Preoperative imaging features				
Number of nodules (1=1)	2.23 (1.53–3.26)	**<0.001**	1.64 (1.06–2.52)	**0.026**
Largest nodule diameter	1.01 (1.00–1.01)	**<0.001**	1.01 (1.00–1.01)	0.076
Tumor burden (≤50%=1)	3.51 (1.76–6.98)	**<0.001**	1.27 (0.28–5.73)	0.759
Macrovascular invasion (no=1)	2.05 (1.37–3.08)	**<0.001**	0.8 (0.39–1.66)	0.553
Portal vein invasion (no=1)	2.41 (1.53–3.79)	**<0.001**	1.85 (0.38–8.93)	0.444
Extrahepatic vascular invasion (no=1)	1.59 (0.74–3.42)	0.236		
Portal vein thrombosis (no=1)	2.4 (1.1–5.21)	**0.022**	0.73 (0.27–2.04)	0.552
Ascites (no=1)	3.77 (1.63–8.71)	**0.001**	1.94 (0.75–5.04)	0.174
BCLC		**<0.001**		0.526
0	1		1	
A	1.37 (.43–4.4)		2.18 (0.61–7.81)	
B	2.95 (.91–9.64)		1.92 (0.42–8.71)	
C	4.11 (1.24–13.65)		n. a.	
Operative data				
Laparoscopic resection (no=1)	0.44 (0.27–0.72)	**0.001**	0.81 (0.43–1.52)	0.514
Operative time (≤180 min =1)	1.35 (0.92–1.99)	0.127		
Operative procedure (minor=1)	1.3 (0.85–1.98)	0.220		
Additional procedures (no=1)	2.07 (1–4.28)	**0.045**	1.4 (0.54–3.62)	0.492
Intraop. blood transfusion (no=1)	2.13 (1.43–3.18)	**<0.001**	1.57 (0.96–2.51)	0.058
Intraop. FFP transfusion (no=1)	1.64 (1.12–2.39)	**0.01**	0.86 (0.48–1.53)	0.609
Pathological data				
R1 resection (no=1)	2.45 (1.19–5.07)	**0.012**	2.22 (0.94–5.23)	0.070
pT category		**<0.001**		0.535
T1	1		1	
T2	2.44 (1.55–3.83)		1.2 (0.53–2.69)	
T3/T4	3.59 (2.15–5.98)		1.7 (0.61–4.75)	
Tumor grading (G1/G2=1)	1.22 (0.78–1.93)	0.386		
MVI (no=1)	2.9 (1.94–4.35)	**<0.001**	2.43 (1.59–3.71)	**<0.001**
Postoperative data				
Intensive care stay (≤1 day=1)	1.52 (0.9–2.55)	0.115		
Hospitalization (≤10 days=1)	1.59 (1.09–2.32)	**0.014**	0.75 (0.44–1.29)	0.303
Postop complications (I/II=1)	2.91 (1.87–4.53)	**<0.001**	2.62 (1.58–4.35)	**<0.001**
PHLF ISGLS (no=1)	1.49 (0.95–2.33)	0.078		
Postop blood transfusion (no=1)	1.61 (1–2.57)	**0.047**	0.79 (0.41–1.51)	0.479
Postop FFP (no=1)	1.93 (1–3.71)	**0.046**	1.31 (0.56–3.09)	0.534

Various parameters are associated with overall survival. A total of 194 cases (89.4%) were included in the multivariable model. For statistically significant parameters (*p*<0.05) bold entries were used

*ALD* alcoholic liver disease, *ALT* alanine aminotransferase, *AP* alkaline phosphatase, *ASA* American Society of Anesthesiologists Classification, *AST* aspartate aminotransferase, *BCLC* Barcelona Clinical Liver Cancer Staging System, *BMI* body mass index, *Excl.* excluded, *FFP* fresh frozen plasma, *GGT* gamma glutamyltransferase, *INR* international normalized ratio, *MELD* model of end-stage liver disease, *MVI* microvascular invasion, *NAFLD* non-alcoholic fatty liver disease, *PHLF* post-hepatectomy liver failure

*AFP was excluded from the multivariable model as the data was only available for 76% of the cohort

**Table 4:** Univariate and multivariable analysis of recurrence-free survival in hepatocellular carcinoma

	Univariate analysis		Multivariable analysis	
	HR (95% CI)	*p* value	HR (95% CI)	*p* value
Demographics				
Gender (male=1)	1.18 (0.78–1.78)	0.426		
Age (years)	1 (0.99–1.01)	0.851		
BMI (≤25 kg/m^2^=1)	1.028 (0.7–1.5)	0.889		
ASA (I/II=1)	1.32 (0.89-1.95)	0.167		
Liver disease		0.436		
ALD	1			
NAFLD	0.74 (0.45–1.22)			
Viral	0.92 (0.55–1.53)			
Cryptogenic/others	0.64 (0.33–1.23)			
Time-to-surgery		0.994		
1–30 days	1			
31–60 days	0.97 (0.61–1.54)			
61–90 days	0.94 (0.54–1.64)			
>90 days	0.93 (0.51–1.71)			
Time-to-surgery (quantitively)	1.01 (1.00–1.01)	0.759		
Preoperative liver function				
MELD score (under 6 = 1)	1.04 (0.95–1.14)	0.387		
Albumin (g/l)	0.9 (0.63–1.29)	0.557		
AFP (μg/l)	1 (0.99–1.01)	0.803		
AST (U/l)	1.01 (1–1.01)	**0.011**	1 (0.99–1.01)	0.913
ALT (U/l)	1.01 (1–1.01)	0.076		
GGT (U/l)	1.01 (1–1.02)	**0.018**	1 (0.99–1.01)	0.341
Bilirubin (mg/dl)	1.4 (0.98–1.99)	0.067		
AP (U/l)	1.01 (1–1.02)	0.206		
Platelet count (/nl)	1 (0.98–1.02)	0.808		
INR	13.04 (1.96–86.9)	**0.008**	19.42 (2.46–153.16)	**0.005**
(mg/dl)	0.77 (0.46–1.26)	0.297		
Hemoglobin (g/dl)	0.99 (0.89–1.1)	0.846		
Child Pugh (A=1)	1.21 (0.69–2.13)	0.504		
Preoperative imaging features				
Number of nodules (1=1)	2.93 (1.99–4.31)	**<0.001**	4.86 (2.19–10.81)	**<0.001**
Largest nodule diameter	1.01 (1–1.01)	**0.001**	1 (0.99–1.01)	0.699
Tumor burden (≤50%=1)	2.68 (1.17–6.14)	**0.015**	0.93 (0.24–3.63)	0.916
Macrovascular invasion (no=1)	2.18 (1.45–3.28)	**<0.001**	0.88 (0.42–1.83)	0.729
Portal vein invasion (no=1)	2.65 (1.68–4.2)	**<0.001**	2.57 (0.91–7.29)	0.075
Extrahepatic vascular invasion (no=1)	2.31 (1.07–4.99)	**0.029**	0.56 (0.20–1.55)	0.266
Portal vein thrombosis (no=1)	5.5 (2.59–11.67)	**<0.001**	2.08 (0.78–5.53)	0.142
Ascites (no=1)	.62 (0.10–3.78)	0.594		
BCLC		**<0.001**		0.066
0	1		1	
A	2.56 (0.62–10.51)		3.73 (0.9–15.48)	
B	5.92 (1.42–24.79)		1.58 (0.31–8.07)	
C	8.22 (1.94–34.89)		2.77 (0.59–13.01)	
Operative data				
Laparoscopic resection (no=1)	0.71 (0.47-1.07)	0.099		
Operative time (≤180 minutes =1)	1.31 (0.9–1.92)	0.161		
Operative procedure (minor=1)	1.35 (0.9–2.03)	0.141		
Additional procedures (no=1)	1.67 (0.73–3.81)	0.218		
Intraop blood transfusion (no=1)	1.26 (0.81–1.97)	0.311		
Intraop FFP (no=1)	1.04 (0.7–1.54)	0.856		
Pathological data				
R1 resection (no=1)	2.46 (1.13–5.32)	**0.018**	2.15 (0.84–5.55)	0.112
pT category		**<0.001**		0.104
T1	1		1	
T2	2.39 (1.54–3.71)		1.31 (0.63–2.70)	
T3/T4	3.87 (2.33–6.43)		2.43 (0.98–6.02)	
Tumor grading (G1/G2=1)	1.21 (0.76–1.92)	0.419		
MVI (no=1)	2.28 (1.55–3.37)	**<0.001**	2.32 (1.51–3.55)	**<0.001**
Postoperative data				
Intensive care stay (≤1 day=1)	1.1 (0.65–1.87)	0.731		
Hospitalization (≤10 days=1)	0.98 (0.66–1.45)	0.918		
Postop complications (I/II=1)	1.15 (0.58–2.29)	0.681		
PHLF ISGLS (no=1)	1.04 (0.63–1.73)	0.870		
Postop blood transfusion (no=1)	0.9 (0.51–1.61)	0.731		
Postop FFP (no=1)	0.58 (0.18–1.82)	0.339		

Various parameters are associated with recurrence-free survival. A total of 183 cases (91.0%) were included in the multivariable model. For statistically significant parameters (*p*<0.05) bold entries were used

*AFP* alpha fetoprotein, *ALD* alcoholic liver disease, *ALT* alanine aminotransferase, *AP* alkaline phosphatase, *ASA* American Society of Anesthesiologists Classification, *AST* aspartate aminotransferase, *BCLC* Barcelona Clinical Liver Cancer Staging System, *BMI* body mass index, *CI* confidence interval, *FFP* fresh frozen plasma, *GGT* gamma glutamyltransferase, *INR* international normalized ratio, *ISGLS* International Study Group of Liver Surgery, *MELD* model of end-stage liver disease, *NAFLD* non-alcoholic fatty liver disease, *PHLF* posthepatectomy liver failure

## Discussion

Although improved therapy options with increased interdisciplinary approaches for patients with HCC have been implemented in the last decades, liver resection remains the first option for patients with early disease stage and preserved liver function [9]. However, liver resection in HCC which is usually accompanied by liver cirrhosis and other co-morbidities requires a notable amount of medical resources ranging from surgical theater to intensive care unit and normal ward capacities [17]. Due to the recent COVID pandemic, medical resources were sparse not only in western countries, but across the globe and usually shifted to treat COVID [18]. Therefore, we investigated the role of TTS in surgically resected HCC patients. Within a large European cohort, we demonstrated that TTS was no risk factor for reduced RFS and OS in HCC patients undergoing curative-intent surgery. Interestingly, we also could not identify major differences in perioperative characteristics of patients with different TTS intervals in our analysis. Furthermore, we determined age, ASA score, preoperative INR, multifocal disease, largest nodule diameter, MVI, and postoperative complications as independent prognostic factors of OS and INR, multifocal disease, and MVI as independent prognostic factors of RFS.

The currently available literature reveals conflicting results regarding the influence of TTS in HCC. While in a retrospective monocentric study by Signal et al. a worse survival due to delayed TTS was observed, a more recent multicentric study of Rao et al. showed no statistical significance of a treatment delay above 90 days on OS of HCC patients [19, 20]. Of note, both studies were not focused on surgically treated patients and included locoregional and systemic therapies across a large disease spectrum. In the cohort of Rao et al., only 31.3% were treated by liver resection, while in the publication of Signal et al. 28% of all patients did undergo surgery demonstrating a limited view on patients with early-stage HCC. Another large study by Govalan et al. demonstrated no association between delay in treatment for HCC and worse OS according to the data of 100,000 patients [21]. Although 38% of the included patients were treated with liver resection, non-curative modalities were also included in this investigation. While profiting from a large dataset, these multicenter datasets do only include a limited number of preoperative characteristics especially detailed tumor staging with associated risk factors, e.g., tumor spread and vascular invasion as well an undetailed view on patients’ performance. Thus, to the best of our knowledge, our study is the first report focusing on TTS in a cohort of surgical patients.

Interestingly, a large systematic review demonstrated a worsened OS after each 4 weeks of delay to definitive surgery in bladder, breast, colon, and head/neck cancer [12]. Regarding other carcinomas of the gastrointestinal tract, a 2020 published study showed an improved OS in pancreatic adenocarcinoma if surgery was conducted within 6 weeks after time of diagnose [22]. For gastric cancer on the other hand, a prolonged time to surgery seems to have no effect on OS according to a recent study [23]. In the case of colorectal liver metastasis undergoing liver resection, a larger monocentric retrospective study displayed a worse OS for patients undergoing liver resection with a time to surgery longer than 6 months [24]. Of note, a part of this cohort underwent neoadjuvant chemotherapy, whereas in our study, patients with any preoperative treatment were excluded to reduce bias in the cohort. Given these different findings for common visceral cancers, it is debatable to shift focus to tumors which are more prone to TTS-related effects due to their inherent malignant potential.

Interestingly, the median TTS was significantly higher during the COVID pandemic compared to the time interval before the COVID pandemic exemplifying the aforementioned shift in medical resources also in our university hospital. As TTS was not associated with OS or RFS in our analysis, it is assumable that this specific delay might not have an influence on long-term outcome. However, this research question must be readdressed and studied in detail after a sufficient follow-up time for these recent patients.

In some circumstances, emergency surgery for HCC is necessary, e.g., because of acute tumor bleeding. Subsequently, these cases were also excluded from our analysis. However, in any other scenario, it seems debatable to delay TTS in the surgical candidates to preoperatively improve the performance status as our results suggest that this might not necessarily impair long-term oncological results. Moreover, in our cohort, a notable part of patients was assessed as ASA > III (65%, 141/217). Moreover, ASA score and postoperative complications were determined as independent factors for reduced OS as also demonstrated in other studies [25]. Thus, using the TTS to improve the patient’s condition prior to surgery appears reasonable. Prehabiliation is a health care intervention prior to surgery comprising lifestyle changes and training resulting in improved nutritional status and physical and mental fitness in the form of a multimodal and usually multidisciplinary concept [26, 27]. Previous meta-analyses already demonstrated reduced hospitalization [28] and complication rates [29] in patients undergoing prehabilitation prior to major abdominal surgery. Prehabilitation strategies include the improvement of aerobic fitness and body composition by physical therapy and correction of malnutrition by professional nutrition interventions as well as reduction of alcohol consumption, support for smoking cessation, and medical interventions to correct anemia as well as psychological support to improve preoperative anxiety, depression, and low self-efficacy [30]. With healthcare funding being a hotly debated subject in western society, structured prehabiliation programs have not widely been implemented. From a cost efficiency perspective, prehabiliation might not be implemented *en masse* but in selected patients benefiting most from preoperative exercise [31]. Given our data, as well as the high prevalence of sarcopenia in HCC and liver cirrhosis, HCC patients might be ideal candidates for structured prehabiliation programs, which is currently also enforced in our department [32].

Besides our primary observation regarding the oncological influence in HCC, we identified several prognostic factors in our cohort which are in line with the literature and indicate comparability of our data to other datasets. MVI has been identified as an important histological parameter and limitational factor for OS and RFS after liver resection and transplantation before [33, 34]. Although examination of suitable preoperative MVI prediction models is becoming more popular in recent years, postoperative histopathological examination currently seems to be the only valid option for proving MVI in HCC at current state [35, 36]. Further we could identify the number of nodules as independent predictor for OS as also commonly known risk factor for reduced OS [37, 38]. Interestingly, number of nodules as preoperative imaging parameter was described as prognostic preoperative imaging markers for appearance of MVI recently [39, 40]. INR has been identified as independent predictor for OS and RFS in our cohort which was also demonstrated in previous studies [41, 42]. Of note, all independent prognostic variables as defined by our multivariable models associated with OS and RFS were not different in the grouped analysis regarding TTS in our patients underlining the validity our results.

As with all retrospective analyses, our study has certainly limitations having to be considered when interpreting the results. Within the monocentric setting of our study, the data reflects the authors’ individual approach to HCC which might be different to clinical standards of other hepatobiliary centers. Also, due to etiological differences, our implications might not be transferable to Asian patients. While the focus of our study was to investigate the influence of TTS in surgically treated patients, we are not able to report on patients dropping from surgical treatment plans due to progression during waiting time as only a small subset of patients was diagnosed in our hepatobiliary center and most of the TTS interval was based on the time from diagnosis to initially presentation to our hepatobiliary unit and not on the waiting time for surgery. However, as HCC is usually slowly progressing which does also explain our findings, it is assumable that the proportion of patients showing a significant disease progression precluding surgical treatment during waiting time might be low. Of note, especially OS appeared numerically higher in patients with short TTS (1–30 days) compared to patients with longer TTS intervals but did not show statistical significance (*p*=0.602). It is debatable whether a statistically significant benefit would be detectable in a larger data set. However, generic cox regression gave no indication for a relevant effect of a shorter TTS and the better result was not replicable in the RFS analysis. Nevertheless, as with all monocentric analysis, our results warrant further investigations in larger, multicentric data sets.

## Conclusion

Notwithstanding the mentioned limitations, we demonstrated that TTS does not influence OS and RFS in patients with HCC who underwent liver resection in curative intent. This finding might be used for prioritizing patients in the scenario of restricted medical resources. Further, our results suggest prehabilitation as important measure to improve short- and long-term outcomes in surgical candidates with HCC.

### Supplementary information

ESM 1 Figure S1: Study cohort

ESM 2

ESM 3

## Abbreviations

ALD: Alcoholic liver disease
ALT: Alanine aminotransferase
ALPPS: Associating liver partition with portal vein ligation for staged hepatectomy
AP: Alkaline phosphatase
ASA: American society of anesthesiologists
AST: Aspartate aminotransferase
BCLC: Barcelona Clinic Liver Cancer
BMI: Body mass index
CPS: Child Pugh Score
CI: Confidence interval
CRP: C-reactive protein
CT: Computed tomography
CUSA: Cavitron Ultrasonic Surgical Aspirator
CVP: Central venous pressure
ECOG: Eastern Cooperative Oncology Group
FFP: Fresh frozen plasma
GCP: Good clinical practice
GGT: Gamma glutamyltransferase
HCC: Hepatocellular carcinoma
ICU: Intensiv care unit
INR: International normalized ratio
ISGLS: International Study Group of Liver Surgery
MELD: Model of end-stage liver disease
MRI: Magnetic resonance imaging
MVI: Microvascular invasion
NAFLD: Non-alcoholic fatty liver disease
OS: Overall survival
PHLF: Posthepatectomy liver failure
RFA: Radiofrequency ablation
RFS: Recurrence-free survival
TACE: Transarterial chemoembolization
TSH: Two-stage hepatectomy
TTS: Time to surgery
UH-RWTH: University Hospital Rheinisch-Westfälische Technische Hochschule
UICC: Union for International Cancer Control

## Declarations

All procedures performed in studies involving human participants were in accordance with the ethical standards of the institutional and/or national research committee and with the 1964 Helsinki declaration and its later amendments or comparable ethical standards. The study was conducted at the UH-RWTH in accordance with the requirements of the Institutional Review Board of the RWTH-Aachen University (EK 22-342).

## References

[1] Sung H, Ferlay J, Siegel RL, Laversanne M, Soerjomataram I, Jemal A (2021). Global Cancer Statistics 2020: GLOBOCAN Estimates of Incidence and Mortality Worldwide for 36 Cancers in 185 Countries. CA Cancer J Clin.

[2] Grandhi MS, Kim AK, Ronnekleiv-Kelly SM, Kamel IR, Ghasebeh MA, Pawlik TM (2016). Hepatocellular carcinoma: from diagnosis to treatment. Surgical Oncology..

[3] Bruix J, Reig M, Sherman M (2016). Evidence-based diagnosis, staging, and treatment of patients with hepatocellular carcinoma. Gastroenterology.

[4] Liu YW, Yong CC, Lin CC, Wang CC, Chen CL, Cheng YF (2020). Liver resection of hepatocellular carcinoma within and beyond the Barcelona Clinic Liver Cancer guideline recommendations: results from a high-volume liver surgery center in East Asia. J Surg Oncol.

[5] Yang B, Zheng B, Yang M, Zeng Z, Yang F, Pu J (2018). Liver resection versus transarterial chemoembolization for the initial treatment of Barcelona Clinic Liver Cancer stage B hepatocellular carcinoma. Hepatol Int.

[6] Cherqui D, Laurent A, Tayar C, Chang S, Van Nhieu JT, Loriau J (2006). Laparoscopic liver resection for peripheral hepatocellular carcinoma in patients with chronic liver disease: midterm results and perspectives. Ann Surg.

[7] Buechter M, Thimm J, Baba HA, Bertram S, Willuweit K, Gerken G (2019). Liver maximum capacity: a novel test to accurately diagnose different stages of liver fibrosis. Digestion.

[8] Muhammad H, Zaffar D, Tehreem A, Ting P-S, Simsek C, Turan I et al (2022) An update on usage of high-risk donors in liver transplantation. J Clin Med [Internet] 11(1) 10.3390/jcm11010215 PMC8746244 35011956

[9] Reig M, Forner A, Rimola J, Ferrer-Fàbrega J, Burrel M, Garcia-Criado Á (2022). BCLC strategy for prognosis prediction and treatment recommendation: the 2022 update. J Hepatol.

[10] Tsilimigras DI, Moris D, Hyer JM, Bagante F, Sahara K, Moro A (2020). Hepatocellular carcinoma tumour burden score to stratify prognosis after resection. Br J Surg.

[11] Arndt V, Doege D, Fröhling S, Albers P, Algül H, Bargou R et al (2022) Cancer care in German centers of excellence during the first 2 years of the COVID-19 pandemic. J Cancer Res Clin Oncol 1-7 10.1007/s00432-022-04407-1 PMC9568964 36241862

[12] Hanna TP, King WD, Thibodeau S, Jalink M, Paulin GA, Harvey-Jones E (2020). Mortality due to cancer treatment delay: systematic review and meta-analysis. Bmj..

[13] (2022) The impact of surgical delay on resectability of colorectal cancer: an international prospective cohort study. Colorectal Dis 24(6):708–726 10.1111/codi.16117 PMC9322431 35286766

[14] Bednarsch J, Tan X, Czigany Z, Wiltberger G, Buelow RD, Boor P et al (2022) Limitations of Nerve fiber density as a prognostic marker in predicting oncological outcomes in hepatocellular carcinoma. Cancers (Basel) 14(9) 10.3390/cancers14092237 PMC9103173 35565366

[15] Bednarsch J, Czigany Z, Heij LR, Amygdalos I, Heise D, Bruners P (2022). The role of re-resection in recurrent hepatocellular carcinoma. Langenbeck’s Arch Surg.

[16] Dindo D, Demartines N, Clavien PA (2004). Classification of surgical complications: a new proposal with evaluation in a cohort of 6336 patients and results of a survey. Ann Surg.

[17] Bednarsch J, Czigany Z, Heise D, Joechle K, Luedde T, Heij L (2021). Prognostic evaluation of HCC patients undergoing surgical resection: an analysis of 8 different staging systems. Langenbeck’s Arch Surg.

[18] Emanuel EJ, Persad G, Upshur R, Thome B, Parker M, Glickman A (2020). Fair allocation of scarce medical resources in the time of Covid-19. N Engl J Med.

[19] Singal AG, Waljee AK, Patel N, Chen EY, Tiro JA, Marrero JA (2013). Therapeutic delays lead to worse survival among patients with hepatocellular carcinoma. J Natl Compr Canc Netw.

[20] Rao A, Rich NE, Marrero JA, Yopp AC, Singal AG (2021). Diagnostic and therapeutic delays in patients with hepatocellular carcinoma. J Natl Compr Canc Netw.

[21] Govalan R, Luu M, Lauzon M, Kosari K, Ahn JC, Rich NE (2022). Therapeutic underuse and delay in hepatocellular carcinoma: prevalence, associated factors, and clinical impact. Hepatol Commun.

[22] Gamboa AC, Rupji M, Switchenko JM, Lee RM, Turgeon MK, Meyer BI (2020). Optimal timing and treatment strategy for pancreatic cancer. J Surg Oncol.

[23] Okuno K, Tokunaga M, Yamashita Y, Umebayashi Y, Saito T, Fukuyo R (2021). Impact of preoperative time interval on survival in patients with gastric cancer. World J Surg.

[24] Chen EY, Mayo SC, Sutton T, Kearney MR, Kardosh A, Vaccaro GM (2021). Effect of time to surgery of colorectal liver metastases on survival. J Gastrointest Cancer.

[25] Delis SG, Bakoyiannis A, Biliatis I, Athanassiou K, Tassopoulos N, Dervenis C (2009). Model for end-stage liver disease (MELD) score, as a prognostic factor for post-operative morbidity and mortality in cirrhotic patients, undergoing hepatectomy for hepatocellular carcinoma. HPB (Oxford).

[26] Wynter-Blyth V, Moorthy K (2017). Prehabilitation: preparing patients for surgery. Bmj.

[27] Cabilan CJ, Hines S, Munday J (2016) The impact of prehabilitation on postoperative functional status, healthcare utilization, pain, and quality of life: a systematic review. Orthopaedic Nursing 35(4) 10.1097/NOR.0000000000000264 27441877

[28] Waterland JL, McCourt O, Edbrooke L, Granger CL, Ismail H, Riedel B (2021). Efficacy of prehabilitation including exercise on postoperative outcomes following abdominal cancer surgery: a systematic review and meta-analysis. Front Surg.

[29] Jain SR, Kandarpa VL, Yaow CYL, Tan WJ, Ho LML, Sivarajah SS et al (2022) The role and effect of multimodal prehabilitation before major abdominal surgery: a systemic review and meta-analysis. World J Surg 10.1007/s00268-022-06761-0 36184673

[30] Bongers BC, Dejong CHC, den Dulk M (2021) Enhanced recovery after surgery programmes in older patients undergoing hepatopancreatobiliary surgery: what benefits might prehabilitation have? Eur J Surg Oncol 47(3 Pt A):551-9 10.1016/j.ejso.2020.03.211 32253075

[31] Kuthiah N (2023). Perioperative prehabilitation. Singapore Med J.

[32] Perisetti A, Goyal H, Yendala R, Chandan S, Tharian B, Thandassery RB (2022). Sarcopenia in hepatocellular carcinoma: current knowledge and future directions. World J Gastroenterol : WJG.

[33] Vitale A, Cucchetti A, Qiao GL, Cescon M, Li J, Ramirez Morales R (2014). Is resectable hepatocellular carcinoma a contraindication to liver transplantation? A novel decision model based on “number of patients needed to transplant” as measure of transplant benefit. J Hepatol.

[34] Mazzaferro V, Llovet JM, Miceli R, Bhoori S, Schiavo M, Mariani L (2009). Predicting survival after liver transplantation in patients with hepatocellular carcinoma beyond the Milan criteria: a retrospective, exploratory analysis. Lancet Oncol.

[35] Tang Y, Xu L, Ren Y, Li Y, Yuan F, Cao M (2022). Identification and validation of a prognostic model based on three MVI-related genes in hepatocellular carcinoma. Int J Biol Sci.

[36] Song D, Wang Y, Wang W, Wang Y, Cai J, Zhu K (2021). Using deep learning to predict microvascular invasion in hepatocellular carcinoma based on dynamic contrast-enhanced MRI combined with clinical parameters. J Cancer Res Clin Oncol.

[37] Goh BK, Chow PK, Teo JY, Wong JS, Chan CY, Cheow PC (2014). Number of nodules, Child-Pugh status, margin positivity, and microvascular invasion, but not tumor size, are prognostic factors of survival after liver resection for multifocal hepatocellular carcinoma. J Gastrointest Surg.

[38] Glantzounis GK, Paliouras A, Stylianidi MC, Milionis H, Tzimas P, Roukos D (2018). The role of liver resection in the management of intermediate and advanced stage hepatocellular carcinoma. A systematic review. Eur J Surg Oncol.

[39] Hong SB, Choi SH, Kim SY, Shim JH, Lee SS, Byun JH (2021). MRI features for predicting microvascular invasion of hepatocellular carcinoma: a systematic review and meta-analysis. Liver Cancer.

[40] Chen S, Wang C, Gu Y, Ruan R, Yu J, Wang S (2021). Prediction of microvascular invasion and its M2 classification in hepatocellular carcinoma based on nomogram analyses. Front Oncol.

[41] Wang Y, Sun K, Shen J, Li B, Kuang M, Cao Q (2019). Novel prognostic nomograms based on inflammation-related markers for patients with hepatocellular carcinoma underwent hepatectomy. Cancer Res Treat.

[42] Mao S, Yu X, Shan Y, Fan R, Wu S, Lu C (2021). Albumin-bilirubin (ALBI) and monocyte to lymphocyte ratio (MLR)-based nomogram model to predict tumor recurrence of AFP-negative hepatocellular carcinoma. J Hepatocell Carcinoma.

[43] Balzan S, Belghiti J, Farges O, Ogata S, Sauvanet A, Delefosse D (2005). The “50-50 criteria” on postoperative day 5: an accurate predictor of liver failure and death after hepatectomy. Ann Surgery.

[44] Rahbari NN, Garden OJ, Padbury R, Brooke-Smith M, Crawford M, Adam R (2011). Posthepatectomy liver failure: a definition and grading by the International Study Group of Liver Surgery (ISGLS). Surgery.

