# Supplementary material for: Time to surgery is not an oncological risk factor in HCC patients undergoing liver resection
Source: Langenbecks Arch Surg. 2023 May 10;408(1):187. doi: 10.1007/s00423-023-02922-4 (PMC10169875; doi:10.1007/s00423-023-02922-4)
Supplement: Supplementary file 1 — Figure S1: Study cohort [file 423_2023_2922_MOESM1_ESM.pdf]

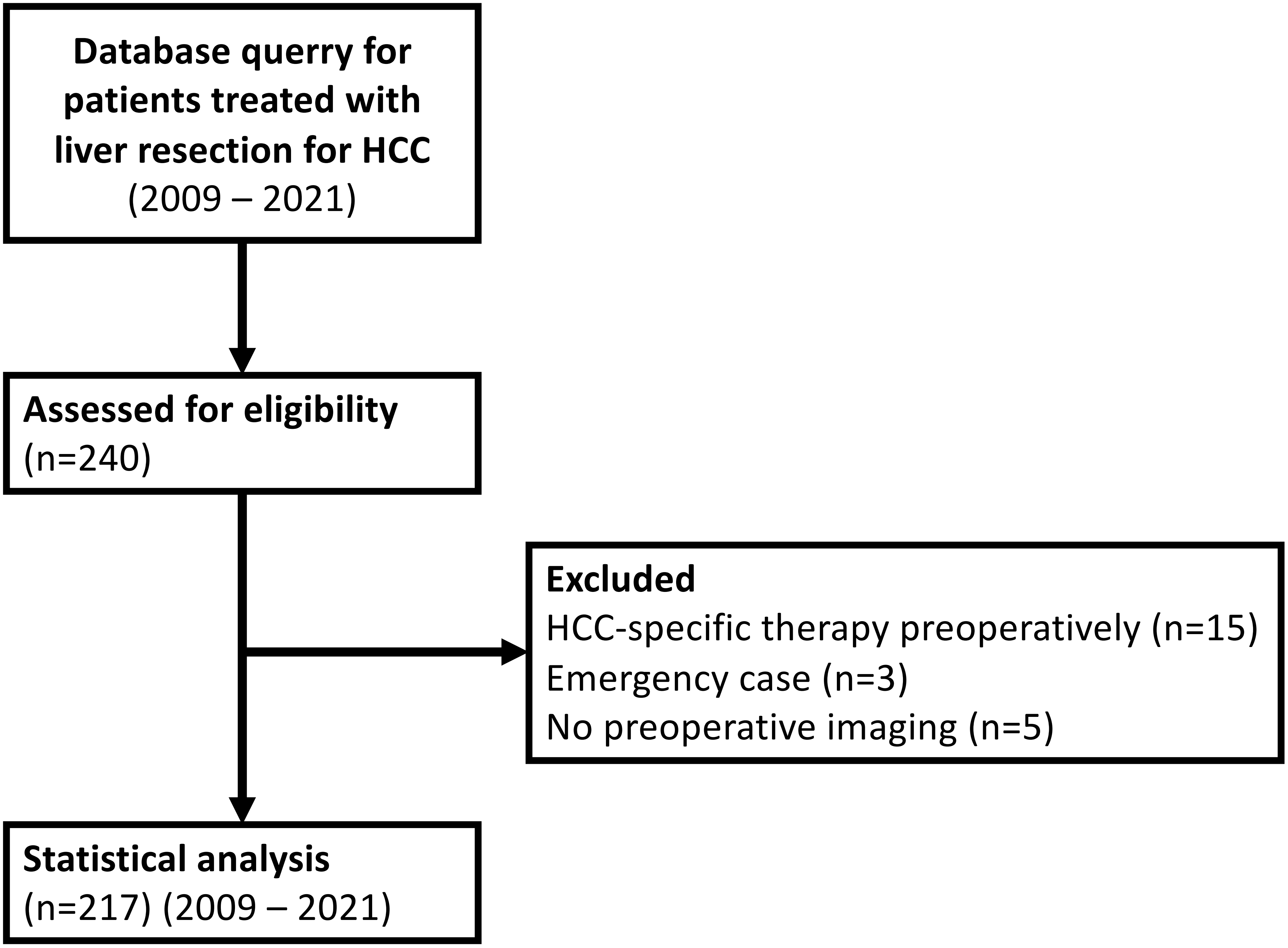
Database querry for patients treated with liver resection for HCC (2009 – 2021)
Assessed for eligibility (n=240)
Excluded
HCC-specific therapy preoperatively (n=15)
Emergency case (n=3)
No preoperative imaging (n=5)
Statistical analysis (n=217) (2009 – 2021)
