# Supplementary material for: Time to surgery is not an oncological risk factor in HCC patients undergoing liver resection
Source: Langenbecks Arch Surg. 2023 May 10;408(1):187. doi: 10.1007/s00423-023-02922-4 (PMC10169875; doi:10.1007/s00423-023-02922-4)
Supplement: Supplementary file 2 — ESM 2 [file 423_2023_2922_MOESM2_ESM.docx]

**Supplementary Table 1: Reason for R1/Rx resection**

| **Variable** | **R1/Rx-Resections (n=12)** |
| --- | --- |
| Intraoperative frozen section with R0 status but final histology with R1 status | 4 |
| No further resection feasible due to impaired liver function | 4 |
| Incomplete removal of tumor-associated venous thrombus | 2 |
| Rx status due to specimen quality | 2 |

Various reasons for R1/Rx resection were observed in the study cohort.
