# Supplementary material for: Time to surgery is not an oncological risk factor in HCC patients undergoing liver resection
Source: Langenbecks Arch Surg. 2023 May 10;408(1):187. doi: 10.1007/s00423-023-02922-4 (PMC10169875; doi:10.1007/s00423-023-02922-4)
Supplement: Supplementary file 3 — ESM 3 [file 423_2023_2922_MOESM3_ESM.docx]

**Supplementary Table 2: Reason for perioperative mortality**

| **Variable** | **Postoperative deceased patients (n=11)** |
| --- | --- |
| Postoperative hemorrhage | 2 |
| Myocardial infarction | 3 |
| Postoperative liver failure | 3 |
| Pulmonary sepsis | 1 |
| Acute pancreatitis | 1 |
| Abdominal sepsis (bile leakage) | 1 |

Various reasons for perioperative mortality were observed in the study cohort.
